# Use of milnacipran in patients suffering from poststroke depression

**DOI:** 10.4103/0019-5545.55099

**Published:** 2009

**Authors:** Harish Arora, Rajdeep Kaur

**Affiliations:** Department of Psychiatry, GGS Medical College, Faridkot, Punjab-151203, India. E-mail: drharisharora06@gmail.comdr.rajdeep@yahoo.co.in

Sir,

It is known that depression is common after stroke. According to a recent report using DSM- IV[1] criteria, the prevalence of major or minor depression among hospitalized patients following a stroke is 22% and 17% respectively.[2]

The etiological relationship between stroke and depression has been investigated in recent years and a biological basis of many cases of post stroke depression has been suggested.[3–5] Patients with post stroke depression are generally less willing to participate in rehabilitation programs and the recovery of activities of daily living is often delayed.

Stroke patients tend to be older and frailer than normal depressed patients and are particularly sensitive to the side effects of the tricyclic antidepressants. The Serotonin-norepinephrine reuptake inhibitor (SNRI), milnacipran, has been reported to be as effective as conventional antidepressants. While having fewer drug interactions and side effects;[6] its use in the treatment of post stroke depression has been investigated in our study.

Fifteen patients with depression whose onset followed a cerebral infarction or hemorrhage (stroke) have been included in the study. All patients were diagnosis for major depressive disorder using DSM-IV criteria. The severity of depression was measured at baseline and after two, four and six weeks using the 21- item Hamilton rating scale for depression.

The patient group comprised of seven males and eight females, age ranged from 45-75 years (mean age 60.8 years). With regard to the type of stroke, nine patients experienced cerebral infarction and six suffered from cerebral hemorrhage. The lesion location was left hemisphere in seven patients, right hemisphere in five patients, and both hemispheres in three patients. Nine patients had motor deficit, five had sensory deficit, two had dysarthria and one had visual field deficit.

Initially, 50 mg /day of milnacipran was administered twice daily in the morning and at night, the dosage was adjusted weekly according to clinical symptoms. The maximum daily dose of milnacipran was in the range of 50-100 mg twice daily. Patients were allowed to take short acting hypnotics when necessary.

One patient dropped out. One complained of headache and yet another of nausea. No other side effect was noticed.

At the end of the study, 85.71% (12/14) of patients completing the study were in remission (a final Hamilton Rating Scale for Depression score of less than seven and no longer meeting the criteria of major depression).

Major improvements were seen in the main Hamilton Rating Scale for Depression items such as depressed mood, work efficiency, psychomotor retardation, psychic anxiety, somatic anxiety, general somatic symptoms, hypochondriasis.

This study demonstrates the efficacy of milnacipran in post stroke depression treatment. Even at a relative low dose of 50-100 mg/day, milnacipran alleviated the symptoms of depression after one/two weeks suggesting that milnacipran has a relatively fast onset of action in these types of patient. Milnacipran was generally well tolerated.

The present study was an open label study and hence it will be necessary to examine more cases and conduct double-blind studies using placebo or other antidepressants. The results from our study suggest that milnacipran may be a promising drug for the treatment of post stroke depression.
